# Book Reviews

**Published:** 1912-05

**Authors:** 


					﻿> BQQK REVIEWS
Professor of Obstetrics, Jefferson Medical College, Phila-
5t|J3lZ •,'lSfe<J¥ota®i®1» °.fe n^>*>«PK	iJltatra-
oloths’f	aBflojM^ders Com-
bnE	Wfe^^M^loO to viraiyzin’J
5	rWWW^	operating
in/?b^5^^dnirtes c^Wflr^e v^^ef^iD^rt^ent part of
recent hteratur^	^^^d^^^ithout this
►aY^oSborltofn vTolmodr-I fnsnoqmi -norn arlf 29713 bboT
nprtBfaTOTajni ns ainoaaiQ oals briE srdsv Iroih
W^rREV-
Dermatology' and Infectious Eruptive Diseases in the Phil-
>ZDA irfWWW^	fan Pyad^a1tqsl
iH jn£ia?2^ndtfd^	235 illus’
isHoO teaibal/ noenaTtal ^nmboP- IfioinilD lo loaaM
Jw§l,2fe9GX>3Si^^rafflWh&,r!Sf1|M|ftlts .^l:P'>Km>ners- By
rs-	® the
re siaor	WeMtWIr1.N-Y-
nimoMsTO	»$;(»!«>
;w notofeWz	>Se‘
» .yamEM' .r^AsSS^I^HWfefSfSs
York, 1912.	.aisBtoobJe rodul
The continued demand which has absorbed several printings
of the first issue of this work, and has now led to the publica-
tion of a new edition, is a fair indication that it has accomplished
the object for which it was created. Its purpose is to furnish
the student and practitioner a brief exposition of Physiology as
a means of quickly reviewing the essential features off the subject.
In this new edition both text nad illustrations have been sub-
jected to a searching revision.
trations. Philadelphia and London: W. B. Saunders Com-
pany, 1911. Cloth, $3.00.
In the present edition the text has been extensively revised
to bring it up to date. The chapter on pellagra has been much
amplified. The article on syphilis has been rewritten to embody
changes made necessary by Ehrlich’s discovery of salvarsan.
Schamberg technique and the Weintraub apparatus seem clumsy
and out of keeping with his other views in regard to this remedy.
A number o.f more expeditious methods have been suggested.
CLINICAL DIAGNOSIS. A Manual of Laboratory Methods.
By James Campbell Todd, M. D., Professor of Pathology,
University of Colorado. Second edition, revised and en-
larged. I2mo, of 469 pages with 164 text-illustrations and
13 colored plates. Philadelphia and London: W. B.
Saunders Company, 1912. Cloth, $2.25 net.
Todd gives the more important laboratory methods of tech-
nical value and also presents an interpretation of the results.
The work is simplified, being designed rather for the student and
practitioner than the experienced laboratory worker.
PRINCIPLES AND PRACTICE OF PHYSICAL DIAGNO-
SIS. By John C. DaCosta, Jr., M. D.. Assistant Pro-
fessor of Clinical Medicine, Jefferson Medical College,
Philadelphia. Second edition, revised/ Octavo of 557
pages, with 225 original illustrations. Philadelphia and
London: W. B. Saunders Company, 1911. Cloth, $3.50 net.
This book deals with the principles of physical diagnosis and
their practical application to the study of thoracic and abdominal
diseases. New matter has been added, chiefly in connection with
the subject of sphygmomanometry, nodal rythm, pleurisy, and
labor atelectasis.
A HANDBOOK OF PRACTICAL TREATMENT. In three
volumes. By 82 eminent specialists. Edited by John H.
Musser, M. D., Professor of Clinical Medicine, University
of Pennsylvania; and A. O. J. Kelly, M. D., Late Assist-
ant Professor of Medicne, University of Pennsylvania.
\ olume III: Octavo of 1095 pages, illustrated. Philadel-
phia and London: W. B. Saunders Company, 1912. Per
volume : Cloth, $6.00 net; Half morocco, $7.50 net.
This well written and well edited work on practice of medi-
cine is probably the best of its kind in English, and for that mat-
ter, in any language. While such works by different writers
are not as cohesive as books written by single authors, it has
many other advantages, such as breadth of view and divergent
views. Modem ideas are incorporated which bring it up to, date
and afford the pratitioner a comprehensive book of reference
which should prove of great value.
NERVOUS AND MENTAL DISEASES. By Archibald
Church, M. D., Professor of Nervous and Mental Diseases
and Mental Jurisprudence in the Northwestern University
Medical School (the Chicago Medical College). Late
Professor of Neurology in the Chicago Polyclinic; Neurol-
ogist to St. Luke’s, Wesley, and Mercy Hospitals; Con-
sulting Neurologist for the Michael Reese Hospital, the
Home for Destitute Crippled Children, etc., and Frederick
Peterson, M. D., Ex-President of the New York State
Commission on Lunacy; Professor of Psychiatry, Colum-
bia University Alienist Hospital; Manager of the Craig
Colony for Epileptics at Sonyea, New York; Ex-President
of the New York Neurologist Society. With 343 illustra-
tions. Seventh edition, thoroughly revised. Philadelphia
and London: W. B. Saunders Company.
This is a carefully prepared text-book written by two men
thoroughly conversant with neurology and psychiatry. Many in-
terpolations and revisions have been made in this edition, and
some chapters largely rewritten. The section on mental diseases
has been wholly rearranged in accordance with the present trend
of classification in America. This seventh edition may be re-
nl ,TZ31ZTl?.WL:0IW^mN5lOOa<lZAH A
gaMic^bia1 ca-u7y'>(ly'iiig'ev'<!Fj'Xiihsir£f,/t,{al a^vaiice itrffle 'ctofnains of
W^Mt <4f?W
-teiaaA aie^I ,.CI .IZ .ylbX J ,O .K bns ; sirifivl’^anna4! io
■TOdkm>X!-«.	New
with
19,1	* IMicS WMr	F	M ’fe WeHfolW volume.
PH&<$i5feW1orlUMl mi^E'Ml^tei^Ptaadelphia
-thorn ^n-£p£^g^j Vdr^’Il¥6^iJ^n lbw brm n'J*hiw Hgw atdT
-Jfim if
-^'h4ZautWf^r^ci«l^iqulfli-
?fi^d fio'^Wdgf* stfefPa	di^titiii-Ss ^kdibWlcd“gdo0f ^fhe
dli)Fe^7b?aA6hies'7'Widg) f/’fg‘f£Huat1'e rIftr;phAtS^d^,v^ j5i¥/fess(5fHrbf
‘A^ldMadVn&llifii’dfi	afi$ ^#ft})k<ftivfeo^actft'i(51igr
WWjf ^ai^tattdh^ W^ft'as 4iei^f^WWdM^<t811eWe
experience of the medical prof^^iftn^&^ito^dre'bSSt ■die#!kiiWrfor
combating each diseases. —He “has -arranged the various diseases
‘^‘pfifi.feeticalK’,1- and^k^^rH^ItfH hafe/gSVeti IfheflbS/t- forifahla^/ffdr
t^^ks Wenpafs>f<w’41-fe>vra?rfOUS>^{ag€h .ifiddcrampications,
				

